# Gonadotropin-releasing hormone alleviates chronic pain-related depression in male mice by rebalancing the anterior cingulate cortex excitatory-inhibitory processes via the protein kinase C/Erb-B2 receptor tyrosine kinase 4 pathway

**DOI:** 10.1093/braincomms/fcag138

**Published:** 2026-04-16

**Authors:** Yanmei Huang, Yunfeng Chen, Xueqin Liu, Yang Xu, Ling Chen, Wenyu Cao, Xiaolin Zhong

**Affiliations:** Department of Metabolism and Endocrinology, the First Affiliated Hospital, Hengyang Medical School, University of South China, Hengyang, Hunan 421001, China; Department of Laboratory Medicine, the First Affiliated Hospital, Hengyang Medical School, University of South China, Hengyang, Hunan 421001, China; Department of Metabolism and Endocrinology, the First Affiliated Hospital, Hengyang Medical School, University of South China, Hengyang, Hunan 421001, China; Department of Metabolism and Endocrinology, the First Affiliated Hospital, Hengyang Medical School, University of South China, Hengyang, Hunan 421001, China; Institute of Neuroscience, Hengyang Medical School, University of South China, Hengyang, Hunan 421001, China; Department of Metabolism and Endocrinology, the First Affiliated Hospital, Hengyang Medical School, University of South China, Hengyang, Hunan 421001, China; Department of Human Anatomy, Hengyang Medical School, University of South China, Hengyang, Hunan 421001, China; Department of Metabolism and Endocrinology, the First Affiliated Hospital, Hengyang Medical School, University of South China, Hengyang, Hunan 421001, China

**Keywords:** chronic pain-related depression, gonadotropin-releasing hormone, anterior cingulate cortex, excitatory-inhibitory balance

## Abstract

Depression is a common comorbidity of chronic pain. Gonadotropin-releasing hormone (GnRH) and its receptor (GnRHR) expressed in the central nervous system are involved in non-reproductive functions. Herein, we aimed to elucidate the role and mechanism of action of GnRH in pain-related depression like behaviour in a mouse model. And we found that both GnRH and GnRHR were down-regulated in the anterior cingulate cortex of mice that were subjected to chronic pain-induced depression with complete Freund’s adjuvant. Specifically, either systemic treatment with GnRH agonists or GnRH overexpression in the anterior cingulate cortex effectively ameliorated the chronic pain-induced depression-like behaviour via GnRHR signalling. Moreover, GnRHR co-localized with both excitatory and inhibitory neurons, and GnRH agonists or overexpressed GnRH rescued the complete Freund’s adjuvant-stimulated imbalance of excitatory-inhibitory neurons in the anterior cingulate cortex. Chemogenetic activation of anterior cingulate cortex neurons reversed GnRH agonist-induced improvement in depression-like behaviour in complete Freund’s adjuvant-treated mice. Furthermore, this specific role of GnRH was dependent on the activation of protein kinase C and Erb-B2 receptor tyrosine kinase 4 signalling pathway. Therefore, our findings indicate that GnRH/GnRHR is involved in the development of chronic pain-related depression, which may through rebalancing the excitatory-inhibitory neurons via the activation of protein kinase C/Erb-B2 receptor tyrosine kinase 4 pathway. Thus, GnRH could be a potential target for the treatment of chronic pain-related depression.

## Teaser

Yanmei Huang *et al.* report that Gonadotropin-releasing hormone alleviates chronic pain-related depression.

## Graphical Abstract

**Graphical Abstract:**
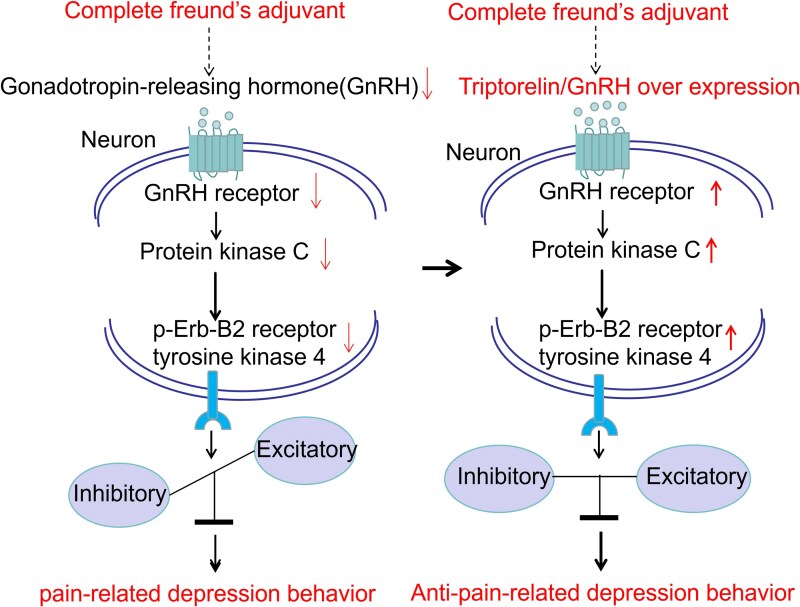

## Introduction

Patients experiencing chronic pain often have accompanying depression. Specifically, pain is a major risk factor for depression, and depression can exacerbate chronic pain and obstruct effective therapies.^1,2^ However, the mechanisms underlying chronic pain-related depression remain unclear. Therefore, it is vital to explore its underlying mechanism and search for a potential strategy to attenuate pain-related depression. Gonadotropin-releasing hormone (GnRH), a decapeptide hypothalamic hormone, is at the head of the neuroendocrine reproductive axis.^3^ GnRH is primarily known to affect the pituitary gland; however, over the last 20 years, several studies have begun to challenge this notion. Increasing evidence indicates that GnRHR is expressed in extra-hypothalamic tissues, such as the cerebral cortex, hypothalamus, hippocampus, cerebellum, spinal cord, neural retina and so on.^4^ It has now become clear that GnRH and GnRHR have diverse biological functions, which may include effects on reproduction, growth, cell survival, tissue repair and renewal, immunomodulation, metabolism, neural function and neuroregeneration.^5,6^ Moreover, studies have demonstrated that GnRH exerts potent neurotrophic, neuroprotective and neuroregenerative effects,^4^ for instance, increasing both outgrowth and length of neurites, accompanied by an increase in neurofilament expression.^7^ GnRH has beneficial effects in patients with brain trauma and spinal cord injuries^4^; it produces antidepressant-like effects in lipopolysaccharide-induced depression in a male mouse model.^8^ These results suggest that the role of GnRH in neurological function parallels its gonadal function, whereas its role in pain-related depression-like behaviour is unclear.

A large body of evidence in animal models suggests that the comorbidity of chronic pain and depression may be related to the involvement of specific brain areas,^9,10^ particularly the hippocampus, amygdala, prefrontal cortex and the anterior cingulate cortex (ACC).^11-13^ The ACC plays a central role in processing pain-related negative effects.^14^ A previous study reported that ACC hyperactivity coincided with depression-like behaviour in mice.^15^ Of note, a proper excitatory-inhibitory (E/I) balance is fundamental for emotional functions in the mammalian cortex.^16^ Excitatory synaptic transmission is driven mainly by glutamatergic synapses, whereas inhibitory synaptic transmission involves γ-aminobutyric acid (GABA) ergic signalling. Especially, GABA-releasing interneurons regulate the activity of excitatory projection neurons, which form the second main class of neurons in the cortex.^17^ Animal models related to depression show reduced expression of the glutamate receptor.^18^ Normalizing the E/I balance could have an antidepressant effect in a mouse model of stress.^19^ Thus, exploring the role of GnRH on the E/I balance of ACC neurons is important to understand pain-related depression-like behaviour.

Erb-B2 receptor tyrosine kinase 4 (ErbB4), which is expressed specifically in GABAergic neurons, has been reported to play a key role in E/I balance. ErbB4 activation maintains GABAergic neuronal activity in the amygdala for fear memory.^20^ ErbB4 variants may cause a greater disruption in the E/I balance,^21^ and ErbB4 inhibition increased spontaneous inhibitory postsynaptic currents in cortical slices.^22^ Mechanistically, ErbB4 decreased postsynaptic currents through GABA-A receptors on inhibitory interneurons through protein kinase C (PKC) activation; the PKC signalling pathway was activated through GnRHR by increased phosphorylation of myristoylated alanine-rich C kinase substrate (MARCKS).^23^ However, the effects of GnRH on ErbB4-mediated E/I balance and whether this process requires PKC have not been studied yet in pain-related depression.

Herein, we aimed to elucidate the role of GnRH in pain-related depression like behaviour in a mouse model. Additionally, we explored the mechanism underlying GnRH function in the ACC.

## Methods

### Animals

Healthy eight-week-old male C57BL/6J mice (obtained from the Hunan SJA Lab Animal Centre of Changsha, Hunan, China) were used in this study. All mice were group-housed (9 mice per cage) in a temperature-controlled (22 ± 2°C) and illumination (12 h light/dark cycles) controlled room, with standard laboratory mouse chow and water provided ad libitum. The experimental protocol was approved by the Animal Care and Use Committee of the University of South China (approval number: 2022usc05xs09) in compliance with the National Institutes of Health Guide for the Care and Use of Laboratory Animals.

### Drugs and treatments

A mouse model of pain-related depression was established by injecting 10 μl of complete Freund’s adjuvant (CFA, Sigma, Shanghai, China) into the plantar right hind paw in mice,^24^ and the same volume of normal saline (NS) was injected into the control group mice. The dosage and method of administration of the GnRH agonist triptorelin (Trip) were according to our previous study.^8^ Briefly, from Days 7 through 21 after CFA or NS injection, the mice were administered an intraperitoneal injection of Trip solution in NS (0.2 mg/kg, MedChemExpress) once a day, whereas the control mice were treated with the same amount of NS (NS group). GnRH antagonist, cetrorelix (CEX, 0.1 mg/kg, Meilunbio); ErbB4 inhibitor, dacomitinib (1 mg/kg, Targetmol); or PKC inhibitor, bisindolylmaleimide I (0.02 mg/kg, Targetmol) were dissolved in NS and injected intraperitoneally on Days 19 through 21 after CFA or NS injection. Twenty-one days after the CFA or NS injection, behavioural tests such as pain threshold, sucrose preference, open-field, forced swim and tail suspension were sequentially performed with a 1-day interval between each test, and the ACC were collected from the experimental group animals. For all behavioural tests, *n* = 9 mice from each group were used, of which *n* = 3 mice were used for the immunofluorescence test and *n* = 6 mice for the western blot test.

### Paw withdrawal threshold

Pain threshold measurements were conducted on Day 21 after CFA injection, as in our previous study.^25^ Before the test, the mice were individually acclimated in plastic compartments with metal mesh bottoms for a 30-min period. The right hind paw was mechanically stimulated using Von Frey filaments; a series of filaments representing different forces, starting from 0.16 g or 2 g, were placed perpendicularly against the plantar of the hind paw of the mice. A positive reaction by the rodent (apparent withdrawal, licking, jumping) was recorded as ‘X’, and a weaker filament was used; a negative reaction was recorded as ‘O’, and a stiffer filament was then applied until we obtained six readings of O and X. The final paw withdrawal threshold was obtained using the formula by Chaplan *et al*.^26^

### Paw withdrawal latency

The hotplate test (Shanghai Xinruan Information Technology Co., Ltd., China) was used to measure the Paw withdrawal latency (PWL). Before the experiment, the mice underwent a 30-min acclimatization period to eliminate explorative behaviour. The hot-plate analgesia meter was activated with the temperature set to 53°C. The hot plate was covered with a layer of transparent plexiglass. Positive responses were identified by the rapid lifting, retracting, or licking of the right hind foot, which was indicative of pain sensation. To avert potential tissue injury, the duration of each trial was limited to 40 s. Each mouse was subjected to this procedure thrice, with the mean of these measurements recorded for analysis.

### Open field test

An exclusive mouse open field platform (40 × 40 × 25 cm^3^) was used for the experiment. The bottom of the box was uniformly divided into 25 equal squares. In the open field experiment, the animals were placed in the inner area and allowed to freely explore for 5 min uninterrupted. The movements of the mice in the open field were carefully recorded using a video camera connected to an automated tracking system (SuperMaze+, Shanghai Xinruan Information Technology Co., Ltd.). The total distance travelled was used as an indicator of their spontaneous locomotor activity.

### Sucrose preference test

All the mice underwent individual housing for a 24-h acclimatization period. Each cage was equipped with a bottle containing 1% sucrose solution. After the completion of the adaptation phase, the experiment was officially initiated. Each mouse was provided with a 100 ml bottle of 1% (w/v) sucrose solution and an equivalent bottle of pure water for consumption. The positions of the two bottles were switched every 12 h to avoid a side preference. After 24 h, the bottles were re-weighed to ascertain the quantity of sucrose solution and water consumed by the mice. The preference for sucrose solution was quantified using the following formula: preference% = ([sucrose consumption]/[sucrose consumption + water consumption] × 100%).

### Tail suspension test

Mice were suspended in the middle of a three-walled rectangular compartment with a climb stopper placed around the tail before applying the tape. A 6-min session was videotaped and analysed for the immobility time in the last 4 min. The behavioural data were subsequently analysed using specialized behavioural analysis software (Shanghai Xinruan Information Technology Co., Ltd.).

### Forced swimming test

Mice were subjected to a swimming test within a plastic cylinder filled with water at a temperature of (25 ± 1)°C. The cylinder measured 25 cm in height and 10 cm in diameter. The mice were gently placed in the apparatus, and their activities were recorded for 6 min. The immobility time in the last 4 min was used as an evaluation index of their depression-like behaviour.

### GnRH enzyme-linked immunosorbent assay

Tissue samples from the ACC of mice across all experimental groups were homogenized in lysis buffer containing phenylmethylsulfonyl fluoride (CWBIO, China). GnRH ELISA kits (CUSABIO, China) were used to quantify the levels of GnRH in mouse ACC tissues. The protocol included adding 100 µL of either the standard solution or the tissue sample to each well, followed by incubation at 37°C for 2 h. Next, 100 µL of the primary antibodies was applied to each well and then incubated at 37°C for an additional hour. After further washing, 100 µL of horseradish peroxidase (HRP)-conjugated secondary antibodies were added to each well and incubated at 37°C for 40 min. The colourimetric reaction was developed in the dark using tetramethylbenzidine as the substrate, and the optical density was measured at 450 nm to determine the concentration of GnRH.

### Stereotactic injection of lentivirus into the ACC

Surgery was performed using a mouse brain stereotactic apparatus (RWD, Shenzhen, China) on Day 7 after CFA or NS injection. Mice were anaesthetized with a dose of 10% sodium pentobarbital intraperitoneally at 50 mg/kg. Microinjection of lentivirus (GnRH overexpression; Lv-GnRH; Shanghai Jikai Gene Chemical Technology Co., Ltd.) and adenovirus (AAV-hM3Dq; Shanghai Jikai Gene Chemical Technology Co., Ltd.) labelled with green fluorescent protein (GFP) were administered into the ACC of both hemispheres. The AAV-hM3Dq (AAV2/9-hSyn-hM3D(Gq)-ER2-P2A) is an AAV-based gene vector using the AAV2/9 serotype, which has high central nervous system transduction efficiency. It employs the human synapsin 1 (hSyn) promoter to specifically express the hM3D(Gq) receptor in neurons, which can be activated by the synthetic ligand clozapine-N-oxide (CNO) to study the effect of neuronal activity on behaviour. For stereotactic injection, anaesthetized mice were fixed on the brain stereotactic apparatus, and small bilateral holes were drilled into the skull following the coordinates: 1.0 mm anterior to the bregma, 0.3 mm lateral to the midline and 1.8 mm in depth. The titre of the GnRH overexpressed virus was 1 × 10^9^ TU/ml, and that of the AAV-hM3Dq virus was 2 × 10^9^ TU/ml. The virus-containing solution (1 μl) was injected at a rate of 0.1 μl per min sequentially into each side of the ACC. Penicillin powder was used to prevent infection. After surgery, the mice were placed in a warm environment until they regained consciousness. Indomethacin Babu ointment was used on the wounds in the mice to reduce postoperative pain in 3 days, and then they were allowed to recover in their home cages for 3 weeks. Behavioural assessments were conducted 2 weeks after the injection.

### Immunofluorescence

Mice were deeply anaesthetized with an overdose of 10% sodium pentobarbital (80 mg/kg) and transcardial perfusion with NS and 4% paraformaldehyde. The brains were quickly dissected and fixed in 4% paraformaldehyde at 4°C overnight. After sequential dehydration in 15% and 30% sucrose solutions, 30 µm coronal sections of the brain were obtained using a cryostat at −22°C. The sections were rinsed thrice with 0.01 M phosphate-buffered saline (PBS) for 10 min each, followed by blocking with 5% donkey serum at room temperature for 2 h. Next, the sections were incubated in a mixture of the primary antibodies, rabbit anti-GnRHR antibody (1:1000, Bioss) and mouse anti-calcium/calmodulin-dependent protein kinase II (CaMKII; 1:500, Proteintech) or mouse anti-glutamate decarboxylase 67 (GAD67; 1:500, Proteintech) or mouse anti-ErbB4 (1:500, Abconal), at room temperature for 2 h, followed by overnight incubation at 4°C. On the following day, the sections were rinsed thrice with 0.01 M PBS for 10 min each, and incubated in a mixture of Alexa 488- or Cy3-labelled donkey anti-rabbit or anti-mouse secondary antibodies (1:250) (Invitrogen, Grand Island, NY, USA) in the dark at room temperature for 2 h. Next, the sections were rinsed again with 0.01 M PBS for 10 min three times. Finally, the slices were mounted with a 4′,6-diamidino-2-phenylindole/anti-fading agent and observed under a fluorescence microscope.

### Western blotting

Following the behavioural assessment, the mice were deeply anaesthetized with an overdose of 10% sodium pentobarbital (80 mg/kg) 1 h after the test. Subsequently, bilateral ACC tissues were quickly excised and preserved at −80°C. Total protein extraction was performed using a radio-immunoprecipitation assay buffer supplemented with protease and phosphatase inhibitors, and protein concentrations were determined using the BCA Assay Kit (CWBIO, China). The protein samples were separated by 10% sodium dodecyl sulfate-polyacrylamide gel electrophoresis and transferred to polyvinylidene difluoride membranes (ISEQ00010, Millipore, Darmstadt, Germany). The membranes were then blocked with 10% skim milk for 2 h, and subsequently probed with the respective primary antibodies (Table 1) by an overnight incubation at 4°C. On the following day, the membranes were equilibrated at room temperature for 2 h and then rinsed with 0.01 M PBS-Tween 20 thrice for 10 min each. Subsequently, they were incubated with the secondary antibody, HRP-conjugated sheep anti-rabbit or anti-mouse IgG (1:2000) for 2 h. Next, the proteins were visualized using the enhanced chemiluminescence method on the ECL imaging system (Tanon-5500), and band intensities were quantified using the ImageJ software. The ratio of optical densities between the target protein bands and the reference protein bands was statistically analysed.

**Table 1:** Primary antibodies used in this study

Antibody	Source	Specificity	kDa	Dilution	Catalog No.
GnRH	ABclonal	Rabbit	10	1:500	MAB5456
GnRHR	Bioss	Rabbit	32	1:500	BS-1464R
ErbB4	Proteintech	Rabbit	147/180	1:1000	19943-1-AP
Phospho-ErbB4	ABclonal	Rabbit	147/180	1:500	AP1521
vGluT1	Proteintech	Rabbit	67	1:1500	5549-1-AP
vGluT2	ABclonal	Rabbit	64	1:2000	A15177
VGAT	ABclonal	Rabbit	57	1:500	A3129
GAD67	Boster	Rabbit	67	1:500	BA0603-2
MARCKS	ABclonal	Rabbit	80	1:500	A0936
Phospho-MARCKS	ABclonal	Rabbit	75	1:500	AP0402
PKCα	ABclonal	Rabbit	77	1:500	A0267
β-Actin	Proteintech	mouse	42	1:2000	6609-1-Ig

Abbreviations: GnRH, gonadotropin-releasing hormone; GnRHR, gonadotropin-releasing hormone receptor; ErbB4, Erb-B2 receptor tyrosine kinase 4; Phospho-ErbB4, Phospho-Erb-B2 receptor tyrosine kinase 4; vGluT1, vesicular glutamate transporter 1; vGluT2, vesicular glutamate transporter 2; VGAT, vesicular γ-aminobutyric acid transporter; GAD67, glutamate decarboxylase 67; MARCKS, myristoylated alanine-rich C kinase substrate; Phospho-MARCKS, phospho-myristoylated alanine-rich C kinase substrate; PKCα, protein kinase C α.

### Statistical analysis

Data analysis was performed using the GraphPad Prism version 8.0 statistical software, developed by GraphPad Software Inc. The exact sample size and test results of each experiment were accorded to the requirements of statistics and shown in the figure legends. The results of the experiments are presented as the mean ± SEM. Any value >2 SD from the group mean was considered an outlier (a criterion decided on before initiating the experiments) and excluded from the analysis. All data complied with the homogeneity of variance test. Initially, significant differences were determined using Student’s *t*-tests for two-group comparisons, and one- or two-ANOVAs, followed by Bonferroni’s *post hoc* testing for multiple comparisons among more than two groups. A *P*-value < 0.05 (*P* < 0.05) was considered statistically significant.

## Results

### GnRH agonist Trip improves GnRHR-dependent pain-related depression-like behaviour

**Figure 1:**
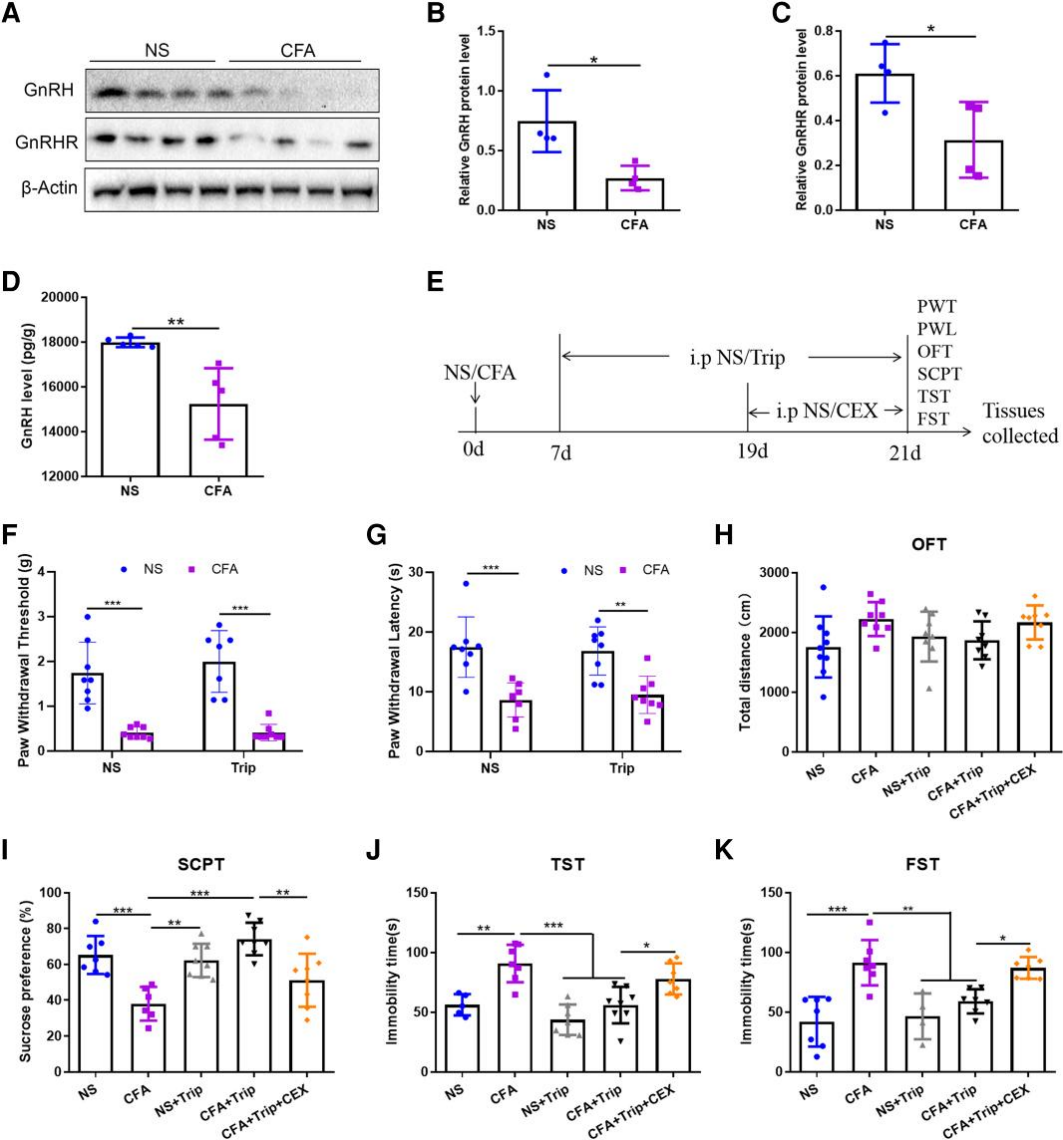
**Expression and role of CFA-induced GnRH/GnRHR in the ACC**. (**A**) Representative western blot immunolabelling of GnRH and GnRHR in the ACC induced by CFA injection, and the original uncropped blots are shown in supplementary Fig. 1. (**B and C**) Semiquantitative analysis of the GnRH and GnRHR proteins relative to β-actin protein. (**D**) GnRH level in the ACC of CFA-induced mice was determined by using ELISA kits. (**E**) The experimental timeline for Trip and CEX treatment after CFA injection. (**F and G**) Effects of Trip on the PWT and PWL in each group. (**H**) Effects of Trip and CEX on the total movement distance of mice in the OFT. (**I**) Effects of Trip and CEX on the sucrose preference rate of 1% sucrose solution in the SCPT. (**J**) Effects of Trip and CEX on the immobility time within 4 min in the TST. (**K**) Effects of Trip and CEX on the immobility time within 4 min in the FST. *n* = 4 mice per group for western blot test, *n* = 5 mice per group for ELISA test and *n* = 5–9 mice per group for behaviour tests and any value >2 SD from the group mean was considered an outlier and excluded from the analysis. Student’s *t*-tests for two-group comparisons, One-way ANOVAs followed by Bonferroni’s *post hoc* testing for multiple comparisons among the groups. Each data point represents the data of one mouse. *P < 0.05, **P < 0.01 and ***P < 0.001. Abbreviations: ACC, anterior cingulate cortex; NS, normal saline; CFA, complete Freund’s adjuvant; GnRH, gonadotropin-releasing hormone; GnRHR, gonadotropin-releasing hormone receptor; Trip, triptorelin; CEX, cetrorelix; ELISA, enzyme-linked immunosorbent assay; PWT, paw withdrawal threshold; PWL, paw withdrawal latency; FST, forced swimming test; OFT, open field test; SCPT, sucrose preference test; Trip, triptorelin; TST, tail suspension test.

Intraplantar administration of CFA is a noxious stimulus commonly used to produce sustained pain in rodents.^27^ In this study, we first observed CFA-induced alteration in the expression of GnRH and GnRHR in the ACC of mice. Western blot demonstrated a significant down-regulation in the protein levels of both GnRH (*t* = 3.420, *P* = 0.0141) and GnRHR (*t* = 2.784, *P* = 0.0318) in the ACC of mice treated with CFA (Fig. 1A–C). Moreover, the GnRH enzyme-linked immunosorbent assay (ELISA) assay also verified that CFA stimulation significantly decreased GnRH levels (*t* = 3.818, *P* = 0.0051) in the ACC of mice (Fig. 1D).

Furthermore, to determine whether GnRH participated in pain hypersensitivity or pain-related depression-like behaviour, we used the GnRH agonist, Trip, and the experimental timeline is shown in Fig. 1E. We found that compared with the NS mice, CFA mice displayed significant mechanical hypersensitivity (*t* = 7.690, *P* < 0.0001) and hot allodynia (*t* = 4.578, *P* = 0.0005) on Day 21 after CFA injection, which could not be alleviated by Trip treatment [paw withdrawal threshold (PWT): *t* = 0.0216, *P* > 0.9999; PWL: *t* = 0.4480, *P* = 0.9984] (Fig. 1F and G). Although no significant differences were observed in the locomotive activity between the groups in the open field test (OFT) (*F*_(4,36)_ = 2.300, *P* = 0.0776) (Fig. 1H), CFA mice displayed depression-like behaviour demonstrated by a significant decrease in the sucrose preference rate in the sucrose preference test (SCPT) (*t* = 4.474, *P* = 0.0009) and an increase in the immobility time in the tail suspension test (TST) (*t* = 4.327, *P* = 0.0016) and forced swimming test (FST) (*t* = 5.748, *P* < 0.0001) when compared with the NS group; Trip treatment reversed the CFA-induced depression-like behaviour (SCPT: *t* = 6.123, *P* < 0.0001; TST: *t* = 4.922, *P* = 0.0003; FST: *t* = 3.761, *P* = 0.0083) (Fig. 1I–K). In addition, we confirmed that the antidepressant effect of the GnRH agonist was dependent on its receptor GnRHR, as we demonstrated that the selective inhibition of GnRHR via CEX intervention abolished the anti-depressive effect of Trip observed in CFA mice (SCPT: *t* = 4.213, *P* = 0.0019; TST: *t* = 3.103, *P* = 0.0424; FST: *t* = 3.254, *P* = 0.0306) (Fig. 1I–K). These behavioural results from pharmacological experiments strongly suggested that GnRH/GnRHR was involved in pain-related depression-like behaviour.

### ACC-specific GnRH overexpression produces antidepressant effects in CFA-treated mice

**Figure 2:**
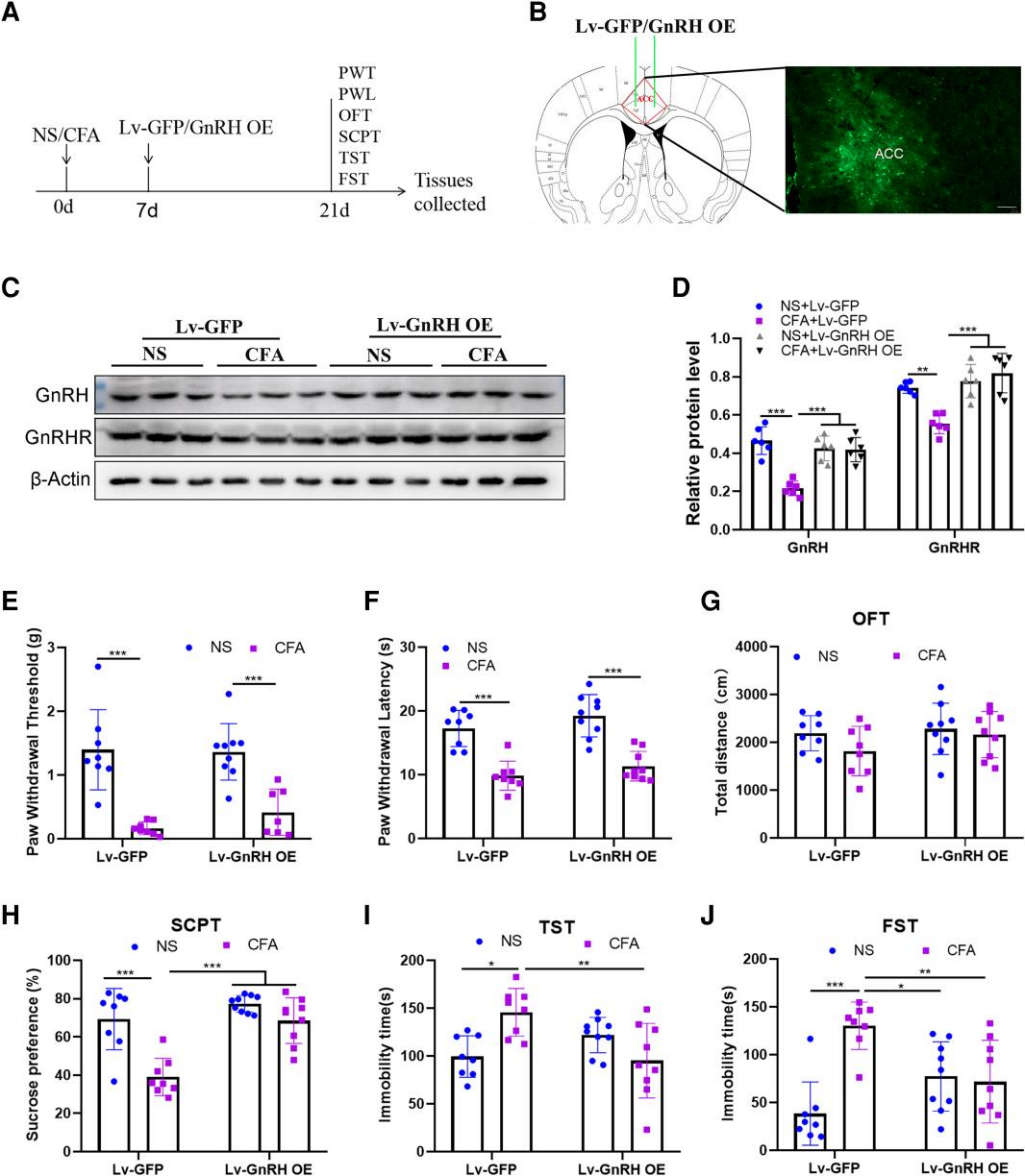
**Role of CFA-induced GnRH overexpression in the ACC**. (**A**) The experimental timeline for GnRH overexpression in the ACC after CFA injection. (**B**) The green fluorescent protein fluorescence demonstrates the site of GnRH overexpression in the ACC, bar = 100 μm. (**C and D**) The verification of GnRH and GnRHR overexpression by western blot and the semiquantitative analysis of the GnRH and GnRH proteins relative to β-actin protein, and the original uncropped blots are shown in supplementary Fig. 2. (**E and F**) Effects of GnRH overexpression on the PWT and PWL. (**G**) Effects of GnRH overexpression on the total movement distance of mice in the OFT. (**H**) Effects of GnRH overexpression on the sucrose preference rate of 1% sucrose solution in the SCPT. (**I**) Effects of GnRH overexpression on the immobility time within 4 min in the TST. (**J**) Effects of GnRH overexpression on the immobility time within 4 min in the FST. *n* = 6 mice per group for western blot test and *n* = 7–9 per group for behaviour tests and any value >2 SD from the group mean was considered an outlier and excluded from the analysis. Two-way ANOVAs followed by Bonferroni’s *post hoc* testing for multiple comparisons among the groups. Each data point represents the data of one mouse. *P < 0.05, **P < 0.01 and ***P < 0.001. Abbreviations: ACC, anterior cingulate cortex; NS, normal saline; CFA, complete Freund’s adjuvant; Lv, lentivirus; GFP, green fluorescent protein; GnRH, gonadotropin-releasing hormone; GnRHR, gonadotropin-releasing hormone receptor; OE, over expression; PWT, paw withdrawal threshold; PWL, paw withdrawal latency; OFT, open field test; SCPT, sucrose preference test; TST, tail suspension test; FST, forced swimming test.

To gain further insight into the role of ACC GnRH in CFA-induced pain-related depression-like behaviour, Lv-GnRH was stereotactically injected into the ACC on Day 7 after CFA injection, and the behaviour test results were examined 2 weeks later (Fig. 2A). The virus injection site is shown in Fig. 2B, and the GnRH overexpression efficiency is indicated by western blot (GnRH: *F*_(1,20)_ = 10.57, *P* = 0.0040; GnRHR: *F*_(1,20)_ = 24.80, *P* < 0.0001) (Fig. 2C and D). Similarly, we found that GnRH overexpression in the ACC had no significant effect on the CFA-induced mechanical hypersensitivity (*t* = 1.590, *P* = 0.6778) and hot hyperalgesia (*t* = 1.601, *P* = 0.6729) in mice (Fig. 2E and F). Moreover, behavioural tests results revealed that GnRH overexpression in the ACC did not affect the locomotor activity (*F*_(1,30)_ = 2.205, *P* = 0.1480) (Fig. 2G); significantly increased the sucrose preference rate (*t* = 7.671, *P* < 0.0001); and shortened the immobility time in CFA-treated mice (TST: *t* = 5.365, *P* = 0.0036; FST: *t* = 4.822, *P* = 0.0096), respectively (Fig. 2H–J). These data indicated that ACC-specific overexpression of GnRH had a significant antidepressant effect.

### GnRH-mediated pain-related depression is associated with the E/I balance of ACC neurons

**Figure 3:**
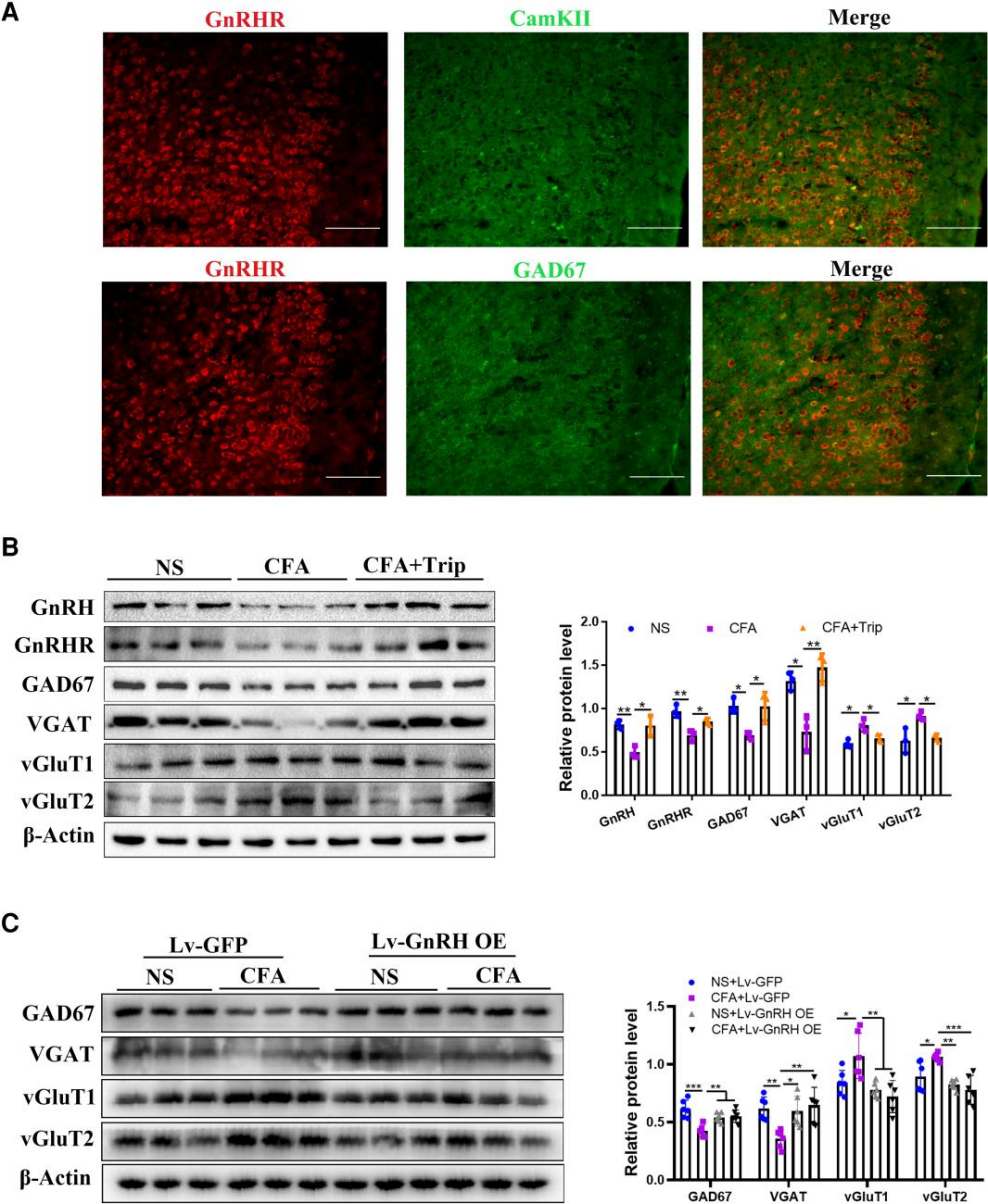
**Effect of GnRH on the E/I balance of ACC neurons induced by CFA injection**. (**A**) The localization of GnRHR (red) with CamKII (green) and GAD67 (green) in the ACC of mice, bar = 200 μm. (**B**) Effects of Trip treatment on the protein levels of GAD67, VGAT, vGluT1 and vGluT2 are demonstrated by western blot and the semiquantitative analysis of these proteins relative to β-actin protein, and the original uncropped blots are shown in supplementary Fig. 3. (**C**) Effects of GnRH overexpression on the protein levels of GAD67, VGAT, vGluT1 and vGluT2 are demonstrated by western blot and the semiquantitative analysis of these proteins relative to β-actin, and the original uncropped blots are shown in supplementary Fig. 4. *n* = 6 per group for western blot. Two-way ANOVAs followed by Bonferroni’s *post hoc* testing for multiple comparisons among the groups. Each data point represents the data of one mouse. *P < 0.05, **P < 0.01 and ***P < 0.001. Abbreviations: ACC, anterior cingulate cortex; NS, normal saline; CFA, complete Freund’s adjuvant; Lv, lentivirus; GFP, green fluorescent protein; GnRH, gonadotropin-releasing hormone; GnRHR, gonadotropin-releasing hormone receptor; OE, over expression; Trip, triptorelin; CaMKII, calcium/calmodulin-dependent protein kinase II; GAD67, glutamate decarboxylase 67; VGAT, vesicular γ-aminobutyric acid transporter; vGluT1, vesicular glutamate transporter 1; vGluT2, vesicular glutamate transporter 2.

To further determine the cell type of GnRHR localization in the ACC neurons, we examined the co-localization of GnRHR with the glutamatergic neuronal marker Calcium-calmodulin (CaM)-dependent protein kinase II (CaMKII) or the GABAergic neuronal marker glutamic acid decarboxylase 67 (GAD67) by using immunofluorescence. As shown in Fig. 3A, a positive result, as indicated by GnRHR-positive cells (red) surrounded by GAD67 or CaMKII-positive signals (green), was observed. These data suggest that the function of both glutamatergic and GABAergic neurons in ACC may be regulated by GnRH.

As the E/I balance is associated with mental disorders,^28^ we focused on the role of GnRH in the E/I balance of ACC neurons. Our results showed that the protein levels of GnRH (*t* = 6.950, *P* < 0.0001) and GnRHR (*t* = 2.975, *P* = 0.0283) were up-regulated after Trip administration in CFA mice. While GAD67 and vesicular γ-aminobutyric acid transporter (VGAT), which are inhibitory neuronal markers, were significantly decreased in the ACC after CFA injection (GAD67: *t* = 5.052, *P* = 0.0004; VGAT: *t* = 6.897, *P* < 0.0001) (Fig. 3B). Conversely, the protein levels of vesicular glutamate transporter (vGluT) 1 and vGluT2, which are excitatory neuronal markers, were significantly increased in the ACC after CFA treatment (vGluT1: *t* = 3.362, *P* = 0.0128; vGluT2: *t* = 3.679, *P* = 0.0035). However, Trip intervention significantly up-regulated GAD67 (*t* = 5.087, *P* = 0.0004) and VGAT (*t* = 8.656, *P* < 0.0001) protein levels, and decreased vGluT1 (*t* = 4.023, *P* = 0.0033) and vGluT2 (*t* = 4.359, *P* = 0.0006) levels in the ACC after CFA injection (Fig. 3B). Additionally, CFA stimulated downregulation of the protein levels of GAD67 (*t* = 8.308, *P* < 0.0001) and VGAT (*t* = 3.798, *P* = 0.0068), and the upregulation of vGluT1 (*t* = 2.959, *P* = 0.0466) and vGluT2 (*t* = 3.136, *P* = 0.0312), while these effects were effectively reversed after ACC-specific GnRH overexpression (GAD67: *t* = 5.592, *P* = 0.0040; VGAT: *t* = 4.212, *P* = 0.0026; vGluT1: *t* = 4.490, *P* = 0.0013; vGluT2: *t* = 5.270, *P* = 0.0002) (Fig. 3C). These data suggested that CFA treatment caused an E/I imbalance in the ACC, which was rescued by the Trip intervention or GnRH overexpression. Thus, GnRH is a key regulator in maintaining the E/I balance of ACC during chronic pain.

### Chemogenetic neuronal activation in the ACC reverses trip-induced improvement in pain-related depression-like behaviour

**Figure 4:**
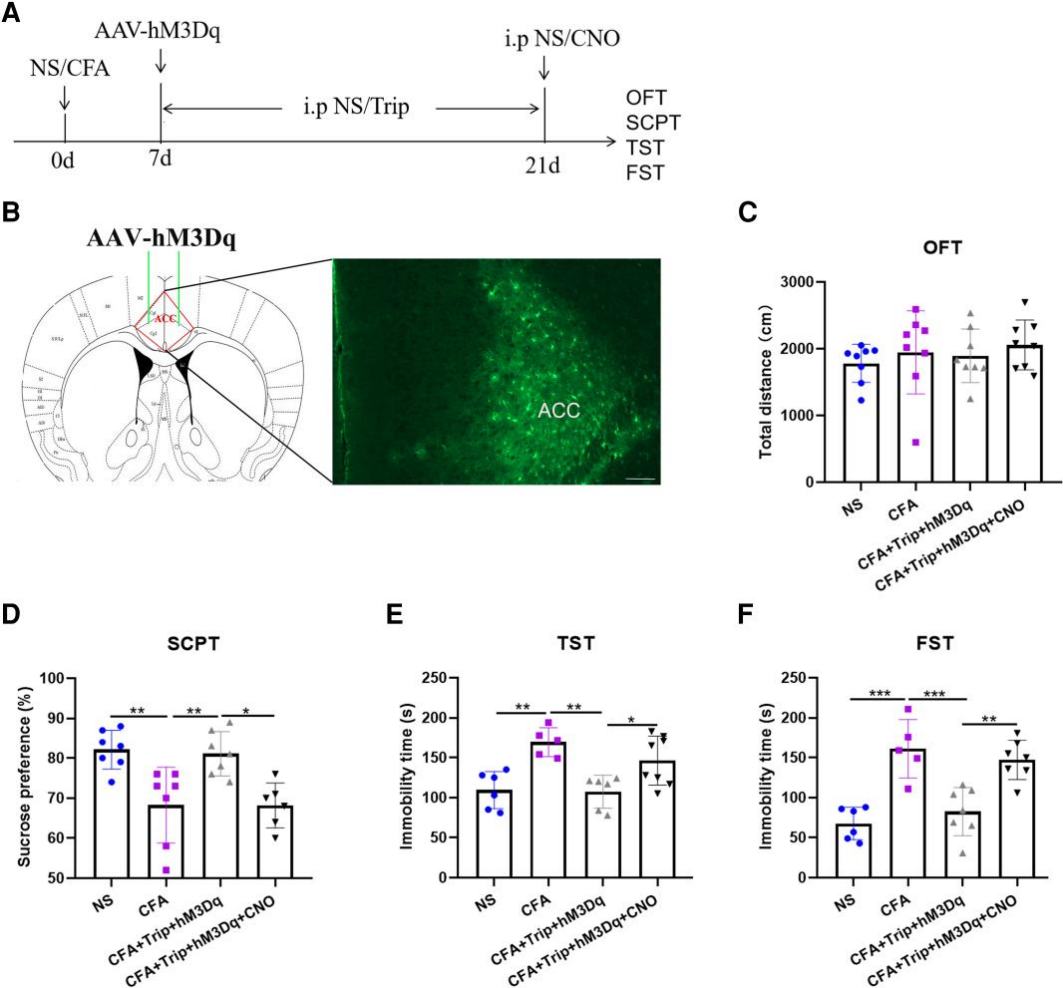
**Effect of ACC neuronal activation on the pain-related depression-like behaviour induced by CFA injection**. (**A**) The experimental timeline for chemogenetic activation of the ACC neurons after CFA injection. (**B**) The green fluorescent protein fluorescence demonstrates the site of AAV-hM3Dq in the ACC, Bar =100 μm. (**C**) Effects of ACC neuronal activation on the total movement distance of mice in the OFT. (**D**) Effects of ACC neuronal activation on the sucrose preference rate of 1% sucrose solution in the SCPT. (**E**) Effects of ACC neuronal activation on the immobility time within 4 min in the TST. (**F**) Effects of ACC neuronal activation on the immobility time within 4 min in the FST. *n* = 5–8 mice per group for behaviour tests and any value >2 SD from the group mean was considered an outlier and excluded from the analysis. One-way ANOVAs followed by Bonferroni’s *post hoc* testing for multiple comparisons among the groups. Each data point represents the data of one mouse. *P < 0.05, **P < 0.01 and ***P < 0.001. Abbreviations: ACC, anterior cingulate cortex; NS, normal saline; CFA, complete Freund’s adjuvant; AAV, adenovirus; hM3Dq, Human muscarinic acetylcholine receptor M3; CNO, Clozapine N-oxide; Trip, triptorelin; GnRH, gonadotropin-releasing hormone; GnRHR, gonadotropin-releasing hormone receptor; OFT, open field test; SCPT, sucrose preference test; TST, tail suspension test; FST, forced swimming test.

Next, to further explore whether the anti-depressive effect of GnRH depended on the normalized E/I balance of ACC, we examined whether manipulated overexcitation in the ACC via chemogenetic neuronal activation would abolish the benefit induced by Trip treatment. AAV-hSyn-hM3D(Gq)-ER2-P2A-EGFP-WPRE-pA was injected into the ACC of mice on Day 7 after CFA injection. Additionally, Trip was injected intraperitoneally from Days 7 through 21 after CFA injection. After 2 weeks of the AAV injection, CNO was injected intraperitoneally 30 min before the behavioural tests to activate the ACC neurons (Fig. 4A and B). As expected, the chemogenetic neuronal activation in the ACC (AAV-hM3Dq + CNO) also had no effects on the locomotor activity in CFA mice (*F*_(3,28)_ = 0.5416, *P* = 0.6578) (Fig. 4C), although the improvement in the pain-related depression-like behaviour in CFA mice caused by Trip treatment was reversed, which was exhibited by the reduced sucrose preference rate (SCPT: *t* = 3.496, *P* = 0.0117) and increased immobility time (TST: *t* = 2.934, *P* = 0.0476; FST: *t* = 4.310, *P* = 0.0019) (Fig. 4D–F) in the CFA + Trip + hM3Dq + CNO mice than in the CFA + Trip + hM3Dq group. Thus, these data suggested that GnRH-induced improvement in pain-related depression-like behaviour required the E/I balance of ACC neurons in the CFA model.

### GnRH maintains the E/I balance in the ACC through ErbB4 in pain-related depression-like behaviour

**Figure 5:**
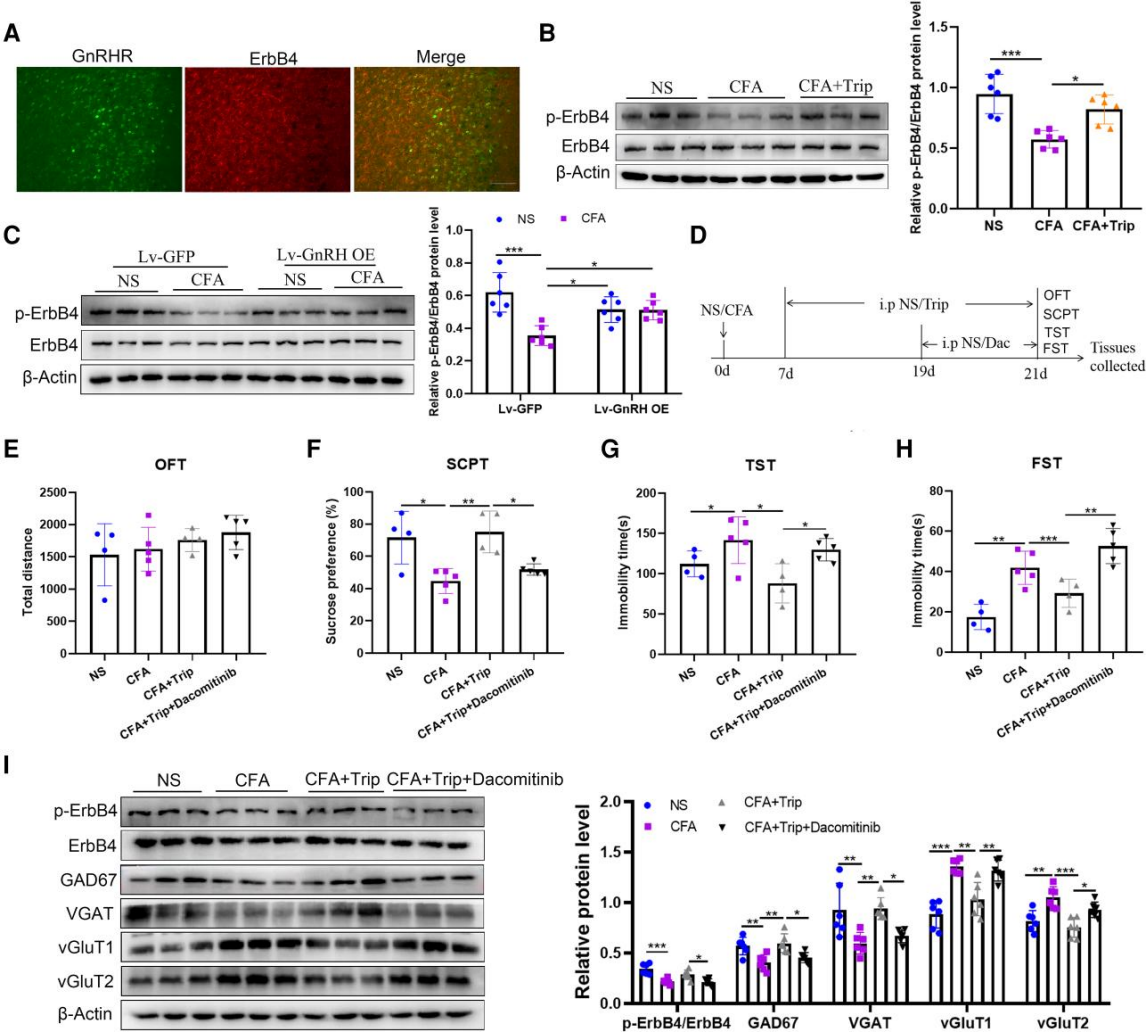
**ErbB4 is the downstream target of GnRH signalling to regulate the E/I balance and pain-related depression-like behaviour induced by CFA injection**. (**A**) The localization of GnRHR (green) with ErbB4 (red) in the ACC of mice, bar = 200 μm. (**B**) Effects of Trip treatment on the protein level of p-ErbB4 are demonstrated by western blot and the semiquantitative analysis of p-ErbB4 relative to ErbB4, and the original uncropped blots are shown in supplementary Fig. 5. (**C**) Effects of GnRH overexpression on the protein levels of p-ErbB4 are demonstrated by western blot and the semiquantitative analysis of p-ErbB4 relative to ErbB4, and the original uncropped blots are shown in supplementary Fig. 6. (**D**) The experimental timeline for Trip and dacomitinib treatment after CFA injection. (**E**) Effects of Trip and dacomitinib treatment on the total movement distance of mice in the OFT. (**F**) Effects of Trip and dacomitinib treatment on the sucrose preference rate of 1% sucrose solution in the SCPT. (**G**) Effects of Trip and dacomitinib treatment on the immobility time within 4 min in the TST. (**H**) Effects of Trip and dacomitinib treatment on the immobility time within 4 min in the FST. (**I**) Effects of Trip and dacomitinib treatment on the protein levels of p-ErbB4, GAD67, VGAT, vGluT1 and vGluT2 are demonstrated by western blot and the semiquantitative analysis of these proteins relative to ErbB4 or β-actin, and the original uncropped blots are shown in supplementary Fig. 7. *n* = 6 mice per group for western blot test and *n* = 4–5 per group for behaviour tests and any value >2 SD from the group mean was considered an outlier and excluded from the analysis. One-way ANOVAs followed by Bonferroni’s *post hoc* testing for multiple comparisons among Trip or dacomitinib treatment groups. Two-way ANOVAs followed by Bonferroni’s *post hoc* testing for multiple comparisons among Lv-GnRH overexpression groups. Each data point represents the data of one mouse. *P < 0.05, **P < 0.01 and ***P < 0.001. Abbreviations: ACC, anterior cingulate cortex; NS, normal saline; CFA, complete Freund’s adjuvant; ErbB4, Erb-B2 receptor tyrosine kinase 4; p-ErbB4, phosphorylated-Erb-B2 receptor tyrosine kinase 4; Lv, lentivirus; GFP, green fluorescent protein; GnRH, gonadotropin-releasing hormone; GnRHR, gonadotropin-releasing hormone receptor; OE, over expression; Trip, triptorelin; Dac, dacomitinib; GAD67, glutamate decarboxylase 67; VGAT, vesicular γ-aminobutyric acid transporter; vGluT1, vesicular glutamate transporter 1; vGluT2, vesicular glutamate transporter 2; OFT, open field test; SCPT, sucrose preference test; TST, tail suspension test; FST, forced swimming test.

Because ErbB4 has been reported to associate with the E/I balance,^20^ we focused on the ErbB4 pathway to investigate the mechanism underlying GnRH in regulating the E/I balance. As shown in Fig. 5A, immunofluorescence revealed completely co-localization of GnRHR and ErbB4 in ACC neurons. Furthermore, western blot assay demonstrated down-regulated of phosphorylated ErbB4 (p-ErbB4) protein level after CFA stimulation of mice (p-ErbB4/ErbB4: *t* = 5.222, *P* = 0.0003), which was significantly reversed by both Trip treatment (p-ErbB4/ErbB4: *t* = 3.441, *P* = 0.0109) (Fig. 5B). In addition, the down-regulated of p-ErbB4 induced by CFA (p-ErbB4/ErbB4: *t* = 7.730, *P* = 0.0001) could be reversed by GnRH overexpression in the ACC (p-ErbB4/ErbB4: *t* = 4.570, *P* = 0.0201) (Fig. 5C).

Next, we investigated whether inhibiting ErbB4 would reverse the improvement in pain-related depression-like behaviour induced by the Trip intervention in CFA mice. ErbB4 inhibitor dacomitinib was injected intraperitoneally on Days 19 through 21 after CFA injection, and control mice were injected with NS (Fig. 5D). Although dacomitinib treatment did not affect the total distance travelled in the OFT (*F*_(3,14)_ = 0.9816, *P* = 0.4295) (Fig. 5E), it significantly reversed the Trip-induced increase in the sucrose preference rate in the SCPT (*t* = 3.247, *P* = 0.0351) and decreased immobility time in the TST (*t* = 2.843, *P* = 0.0782) and FST (*t* = 4.496, *P* = 0.0030) in CFA mice (Fig. 5F–H). Furthermore, western blot also revealed that dacomitinib intervention reversed the effects of Trip on the expression of p-ErbB4/ErbB4 (*t* = 3.090, *P* = 0.0346), GAD67 (*t* = 3.156, *P* = 0.0298), VGAT (*t* = 2.999, *P* = 0.0426), vGluT1 (*t* = 3.984, *P* = 0.0044) and vGluT2 (*t* = 3.155, *P* = 0.0299) (Fig. 5I). These findings indicated that GnRH maintained the E/I balance of ACC, probably through phosphorylated ErbB4.

### GnRH-mediated activation of ErbB4 is associated with the PKC pathway in the ACC of mice with pain-related depression-like behaviour

**Figure 6:**
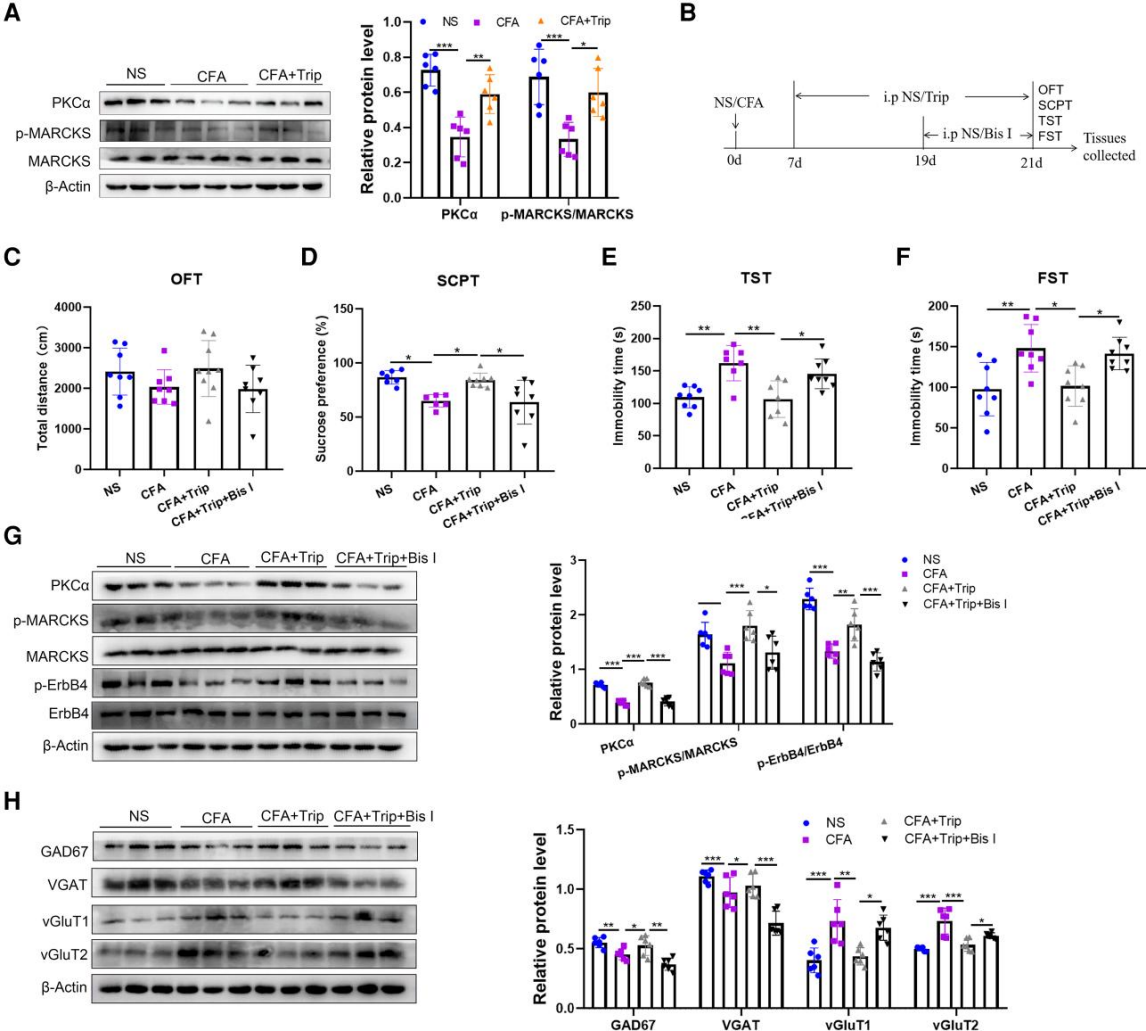
**GnRH activates ErbB4 via the PKC pathway in the ACC of mice after CFA injection**. (**A**) Effects of Trip treatment on the protein levels of PKCα and p-MARCKS are demonstrated by western blot and the semiquantitative analysis of PKCα and p-MARCKS relative to β-actin and MARCKS, and the original uncropped blots are shown in supplementary Fig. 8. (**B**) The experimental timeline for Trip and bisindolylmaleimide I treatment after CFA injection. (**C**) Effects of Trip and bisindolylmaleimide I treatment on the total movement distance by mice in the OFT. (**D**) Effects of Trip and bisindolylmaleimide I treatment on the sucrose preference rate of 1% sucrose solution in the SCPT. (**E**) Effects of Trip and bisindolylmaleimide I treatment on the immobility time within 4 min in the TST. (**F**) Effects of Trip and bisindolylmaleimide I treatment on the immobility time within 4 min in the FST. (**G**) Effects of Trip and bisindolylmaleimide I treatment on the protein levels of PKCα, p-MARCKS and p-ErbB4 were demonstrated by western blot and the semiquantitative analysis of these proteins relative to β-actin, MARCKS and ErbB4, and the original uncropped blots are shown in supplementary Fig. 9. (**H**) Effects of Trip and bisindolylmaleimide I treatment on the protein levels of GAD67, VGAT, vGluT1 and vGluT2 demonstrated by western blot and the semiquantitative analysis of these proteins relative to β-actin, and the original uncropped blots are shown in supplementary Fig. 10. *n* = 6 mice for western blot test and *n* = 6–9 mice per group for behaviour tests and any value >2 SD from the group mean was considered an outlier and excluded from the analysis. One-way ANOVAs followed by Bonferroni’s *post hoc* testing for multiple comparisons among the groups. Each data point represents the data of one mouse. *P < 0.05, **P < 0.01 and ***P < 0.001. Abbreviations: ACC, anterior cingulate cortex; NS, normal saline; CFA, complete Freund’s adjuvant; ErbB4, Erb-B2 receptor tyrosine kinase 4; p-ErbB4, phosphorylated-Erb-B2 receptor tyrosine kinase 4; GnRH, gonadotropin-releasing hormone; GnRHR, gonadotropin-releasing hormone receptor; PKCα, protein kinase C α; MARCKS, myristoylated alanine-rich C kinase substrate; p-MARCKS, phosphorylated-myristoylated alanine-rich C kinase substrate; Trip, triptorelin; Bis I, bisindolylmaleimide I; GAD67, glutamate decarboxylase 67; VGAT, vesicular γ-aminobutyric acid transporter; vGluT1, vesicular glutamate transporter 1; vGluT2, vesicular glutamate transporter 2; OFT, open field test; SCPT, sucrose preference test; TST, tail suspension test; FST, forced swimming test.

GnRHR belongs to the G protein-coupled receptor family, and its stimulation activates PKC.^23^ PKC is involved in ErbB4 activation after GnRH treatment.^29^ Our results revealed significantly reduced PKCα protein level (*t* = 6.275, *P* < 0.0001) and p-MARCKS/MARCKS (*t* = 4.637, *P* = 0.0010) in the ACC of CFA mice, which were reversed by the Trip intervention (PKCα: *t* = 4.023, *P* = 0.0033; p-MARCKS/MARCKS: *t* = 3.483, *P* = 0.0100) (Fig. 6A).

Next, to further explore whether GnRH-mediated ErbB4 activation was PKC-dependent, PKC inhibitor, bisindolylmaleimide I, was injected intraperitoneally from Days 19 through 21 after CFA injection, and control mice were injected with NS (Fig. 6B). As expected, bisindolylmaleimide I intervention did not influence the locomotor activity in CFA mice (*F*_(3,29)_ = 1.617, *P* = 0.2069) (Fig. 6C), although it significantly reversed the Trip-induced improvement in the depression-like behaviour in CFA mice (SCPT: *t* = 3.463, *P* = 0.0112; TST: *t* = 3.165, *P* = 0.0236; FST: *t* = 2.950, *P* = 0.0382) (Fig. 6D–F).

In addition, western blot assay revealed that bisindolylmaleimide I treatment significantly decreased the Trip-induced increase in the expression of PKCα (*t* = 9.888, *P* < 0.0001), p-MARCKS/MARCKS (*t* = 3.349, *P* = 0.0192) and p-ErbB4/ErbB4 (*t* = 5.691, *P* < 0.0001) in CFA mice (Fig. 6G). Furthermore, we found that bisindolylmaleimide I treatment reversed the effects of Trip on the expression of GAD67 (*t* = 4.551, *P* = 0.0012), VGAT (*t* = 6.970, *P* < 0.0001), vGluT1 (*t* = 3.374, *P* = 0.0181) and vGluT2 (*t* = 4.754, *P* = 0.0151) (Fig. 6H). All these data fully demonstrated that GnRH eliminated chronic pain-related depression by rebalancing the E/I of ACC neurons, possibly through the activation of PKC/ErbB4 pathway.

## Discussion

Pain-related depression is one of the most important mental illnesses accompanying pain.^30^ In our current study, we found that the expression of GnRH and GnRHR was decreased in the ACC of a mouse model of CFA-induced depression. GnRH agonist treatment or GnRH overexpression in the ACC eliminated CFA-induced pain-related depression-like behaviour. These benefits of GnRH may be through the rebalancing of the E/I of the ACC neurons, which is dependent on the activation of the PKC/ErbB4 pathway. Therefore, the present study supports that GnRH may serve as a promising prevention target for pain-related depression-like behaviour.

GnRH is secreted from hypothalamic GnRH neurons and stimulates anterior pituitary gonadotrophs to synthesize and secrete the gonadotropins, luteinizing hormone and follicle-stimulating hormone.^31^ A previous study demonstrated the occurrence of GnRH in the entire hypothalamus, olfactory bulb, frontoparietal cortex, preoptic area-anterior hypothalamus and medial basal-posterior hypothalamus.^32^ The distribution and activity of GnRH/GnRHR outside of the hypothalamus, as well as its decreased expression in mouse models of systemic inflammation and ageing,^8,33^ triggered our interest in its possible involvement in pain-related depression. The ACC is involved in the comorbid chronic pain and pain-related depression.^34,35^ Herein, we found that both GnRH and GnRHR decreased significantly in the ACC of mice treated with CFA. Although our previous study identified reduced hippocampal GnRH and GnRHR in a mouse model of systemic inflammation,^8^ we did not observe these changes in the present study (data not shown). We consider that these differences might be due to the different animal model used. Regardless, our data further corroborated ACC as a convergent point of chronic pain and depression.

Subsequently, to verify whether the GnRH/GnRHR system in ACC would be involved in the development of pain hypersensitivity and chronic pain-related depression, we systematically treated the mice with GnRH agonists or overexpressed GnRH in the ACC. GnRH analogues are used extensively to treat male and female infertility and sex hormone-dependent cancers, such as prostate cancer.^36^ Our present study used the GnRH agonist Trip to stimulate GnRH signalling. Although the CFA-induced mechanical hypersensitivity and hot hyperalgesia in mice were unaffected by Trip treatment, notably, CFA-induced depression-like behaviour was prevented. Conversely, systematic injection of the GnRHR antagonist CEX blocked the anti-depression-like effect of the GnRH agonist, which further strengthens the notion that GnRH-GnRHR interaction is involved. Furthermore, to exclude the potential peripheral effect of GnRH, GnRH was overexpressed in the ACC in a cohort of animals, and notably, this effectively alleviated the CFA-induced depression-like behaviour. These results suggest that the ACC GnRH/GnRHR system is involved in the development of chronic pain-related depression. Therefore, GnRH may be a new target for the treatment of chronic pain-related depression.

The excitability and inhibition of neurons are in a balanced state under physiological conditions, although this balance can be disrupted in pathological conditions.^37^ In the mammalian cortex, an E/I balance is fundamental for its functions.^17^ Pharmacological, chemogenetic and optogenetic approaches for disrupting the E/I balance induce impairments in a range of behaviours.^38^ A previous study demonstrated that depression-like behaviour was associated with the E/I imbalance in stress-sensitive brain regions.^39^ Several studies using multiple pain models have provided evidence of the involvement of alterations in the balance of E/I neurotransmission in the ACC.^40,41^ We speculated that the cellular and molecular mechanisms for GnRH/GnRHR to regulate chronic pain-related depression might be related to the balance of E/I neurotransmission in the ACC. Having determined the co-localization of GnRHR with both the glutamatergic neuronal marker CaMKII and the GABAergic neuronal marker GAD67, we then examined whether the activation of GnRH/GnRHR would affect the E/I neurotransmission in the ACC. In line with a previous study, we found down-regulation of inhibitory neuronal markers such as GAD67 and VGAT, and significant upregulation of excitatory neuron markers such as vGluT1 and vGluT2 in the ACC, which suggested that CFA indeed induced the E/I imbalance in the ACC neurons in mice. We demonstrated that both Trip treatment and ACC-specific overexpression of GnRH rebalanced the E/I of ACC neurons, indicated by a reversal of the aforementioned expression patterns of inhibitory and excitatory neuronal markers. Additionally, we observed that chemogenetic activation of ACC neurons blocked the anti-depression-like effect of the GnRH agonist induced by CFA stimulation. Thus, we concluded that GnRH regulation of chronic pain-related depression-like behaviour may depend on the E/I balance of ACC neurons.

The above results prompted us to investigate the signalling pathway underlying the GnRH-mediated E/I balance in the ACC, focusing on ErbB4, a receptor tyrosine kinase that incorporates an epidermal growth factor domain.^42^ The majority of ErbB4-positive cells in the cortex, hippocampus and amygdala are GABAergic interneurons.^43^ Previous investigations have revealed that ErbB4 signalling dynamically modulates GABA release, influencing pyramidal neuron activity,^44^ synaptic plasticity,^45^ and the E/I balance.^46^ Moreover, ErbB4 has been identified as an important susceptibility gene for depression.^47^ Interestingly, our present study showed that GnRHR and ErbB4 were co-expressed in interneurons in the mouse ACC. We demonstrated that inhibition of ErbB4 by dacomitinib significantly blocked the anti-depression-like behaviour and reversed the E/I balance induced by GnRH agonist treatment in CFA mice, which leads us to propose that ErbB4 might be a downstream target of GnRH signalling to regulate the E/I balance.

As a G protein-coupled receptor, GnRHR activated a series of downstream signalling pathways, such as PKC, extracellular signal-regulated kinase (ERK), c-Jun N-terminal kinase (JNK) and protein kinase B (AKT).^48,49^ A previous study has demonstrated that the neuronal hyperexcitability of ACC involves PKC activation and a downregulation of hyperpolarization-activated/cyclic nucleotide-gated (HCN) in the early phase of neuropathic pain.^50^ Additionally, another study has demonstrated that ErbB4-regulated GABAergic inhibitory currents require PKC activation.^51^ A recent study clearly showed that annexin A1 expression was also stimulated by GnRH through PKC in LβT2 gonadotrope cells.^52^ However, whether GnRH/GnRHR stimulation-induced ErbB4 activation is dependent on PKC has never been elucidated. Herein, we found that the down-regulated expression of PKCα and p-MARKS in CFA mice was reversed by Trip treatment. Moreover, inhibition of PKC by its inhibitor bisindolylmaleimide I blocked the effects of Trip on pain-related depression-like behaviour and the E/I balance of ACC neurons. These results indicate that PKC is upstream of ErbB4 in the case of GnRH signalling stimulation in pain-related depression-like behaviour.

### Strengths and limitations

The strengths of this study include we demonstrated GnRH and its receptor expressed in ACC are involved in pain-related depression like behaviour functions. Trip, which is an agonist of GnRH, has been used extensively to treat male and female infertility, and sex hormone-dependent cancers; notably, it may also be a potential drug for the treatment of chronic pain-related depression.

However, there are some limitations in our present study. First, as for the detection of neuronal excitability and inhibition, electrophysiological recordings are not obtained in our present study. Secondly, since ACC is interconnected with other pain-related brain areas to regulate pain and pain-related emotional behaviour, our current design cannot exclude broader network contributions. Thirdly, systemic drug administration does not only reflect the role of ACC, but it may also be the combined effect of ACC and other broader network contributions. Fourthly, E/I imbalance mediates pain pathogenesis, but the mechanism about GnRH improving E/I balance in ACC did not affect pain hypersensitivity was not elucidated in this study. Fifthly, due to the thickness of our brain slices and the non-specific background of staining, we did not quantify the proportion of GnRHR and ErbB4 overlapping cells. Lastly, GnRH/GnRHR mediates glutamatergic and GABAergic signalling, but our subsequent experiments focus on GABAergic function, we haven't explained whether the regulation of glutamatergic signalling is direct or indirect in our current study.

## Conclusion

Our present study demonstrated that GnRH/GnRHR played a key role in maintaining the E/I balance of ACC neurons, resulting in anti-depression-like behaviour induced by chronic pain. Furthermore, the underlying mechanism may involve activation of the downstream PKC/ErbB4 pathway by the GnRH signalling cascade. These data provide new insights that GnRH may represent a new molecular target for the treatment of depression associated with chronic pain.

## Supplementary Material

fcag138_Supplementary_Data

## Data Availability

The data are available on request from the corresponding author. The data are not publicly available due to privacy or ethical restrictions.
