# Supplementary material for: Gonadotropin-releasing hormone alleviates chronic pain-related depression in male mice by rebalancing the anterior cingulate cortex excitatory-inhibitory processes via the protein kinase C/Erb-B2 receptor tyrosine kinase 4 pathway
Source: Brain Commun. 2026 Apr 16;8(2):fcag138. doi: 10.1093/braincomms/fcag138 (PMC13126666; doi:10.1093/braincomms/fcag138)
Supplement: fcag138_Supplementary_Data [file fcag138_supplementary_data.pdf]

GnRH improves chronic pain-related depression by re-balancing anterior cingulate cortex excitatory/inhibitory via PKC/ErbB4 pathway

Yanmei Huang[1,2,#], Yunfeng Chen[1,#], Xueqin Liu[1], Yang Xu[3], Ling Chen[1], Wenyu Cao[4,*], Xiaolin Zhong[1,*]

1. Department of Metabolism and Endocrinology, The First Affiliated Hospital, Hengyang Medical School, University of South China, Hengyang, 421001, Hunan, China
2. Department of Laboratory Medicine, The First Affiliated Hospital, Hengyang Medical School, University of South China, Hengyang, 421001, Hunan, China
3. Institute of Neuroscience, Hengyang Medical School, University of South China, Hengyang, 421001, Hunan, China
4. Department of Human Anatomy, Hengyang Medical School, University of South China, Hengyang, 421001, Hunan, China

[#]Yanmei Huang and Yunfeng Chen contributed equally to this work.

[*] Corresponding authors:

Xiaolin Zhong

Department of Metabolism and Endocrinology, The First Affiliated Hospital, Hengyang Medical School, University of South China, Hengyang, 421001, Hunan, China. zhxl520@usc.edu.cn

Wenyu Cao

Department of Human Anatomy, Hengyang Medical School, University of South China, Hengyang, 421001, Hunan, China. cwy@usc.edu.cn

Uncropped/unedited images for all blots and gels

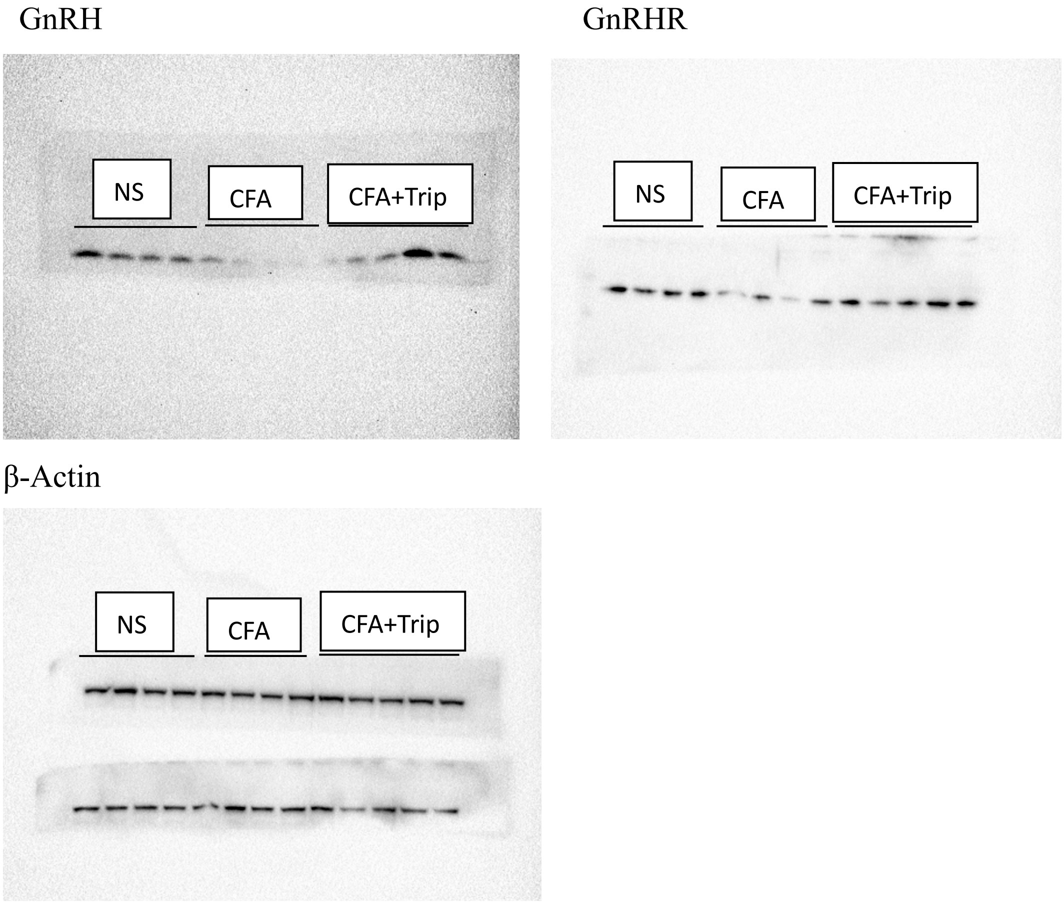

Supplementary Figure 1: Uncropped blots for Figure 1.A

GnRH

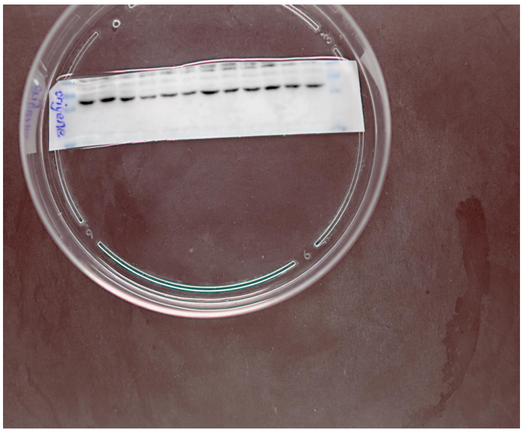

GnRHR

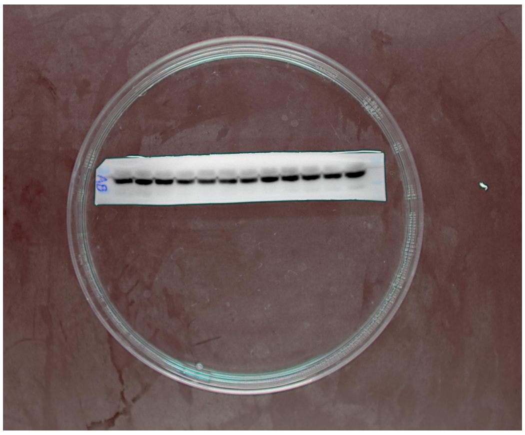

β-Actin

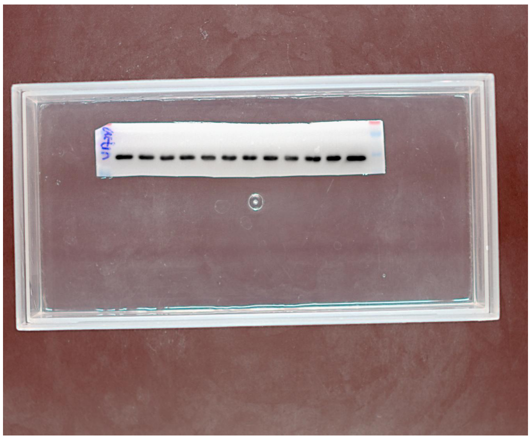

Supplementary Figure 2: Uncropped blots for Figure 2.C

GnRH

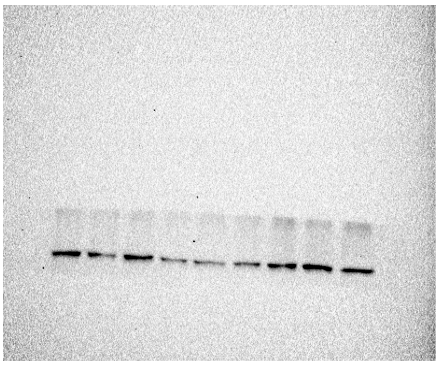

GnRHR

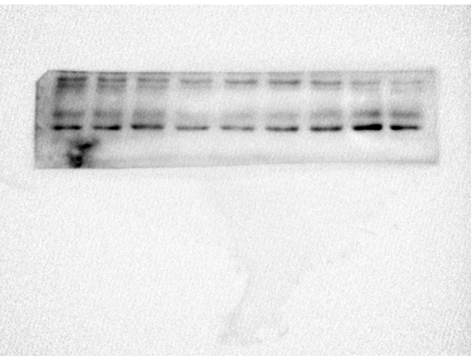

GAD67

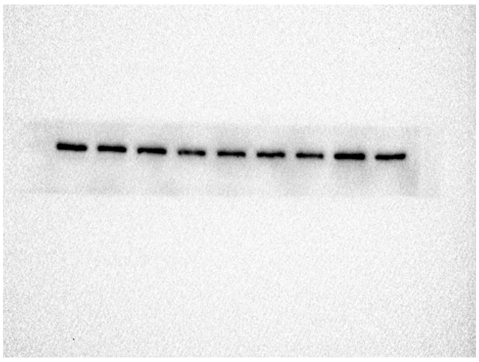

VGAT

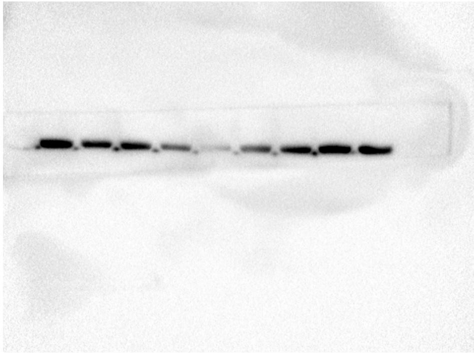

VGluT1

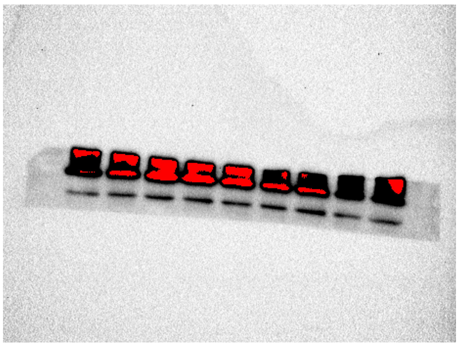

VGluT2

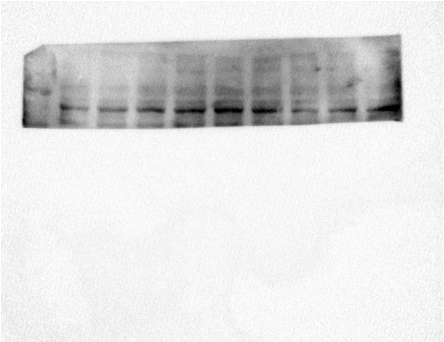

β-Actin

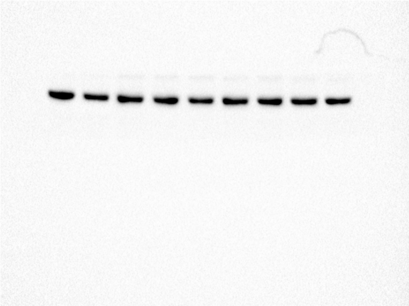

Supplementary Figure 3: Uncropped blots for Figure 3.B

GAD67

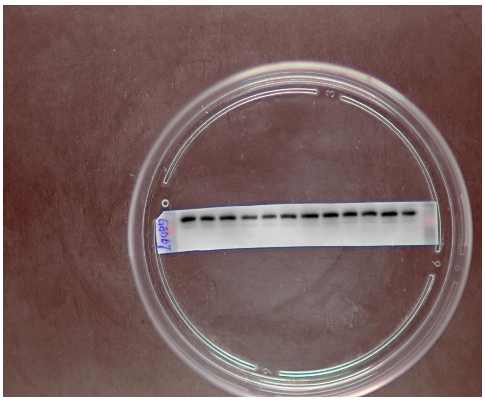

VGAT

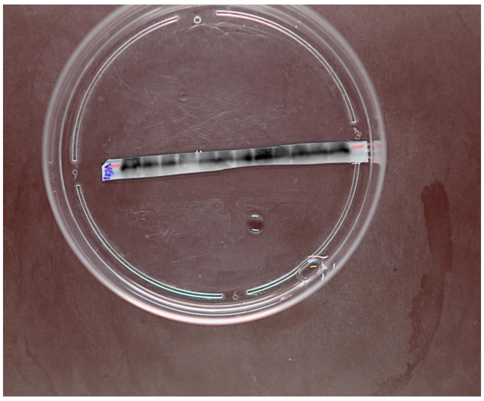

VGluT1

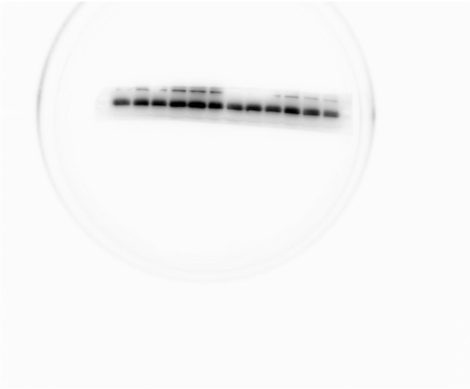

VGluT2

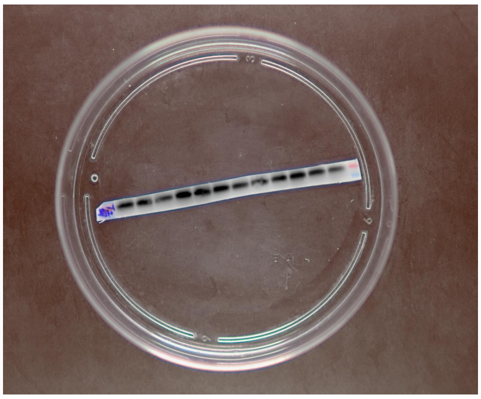

β-Actin

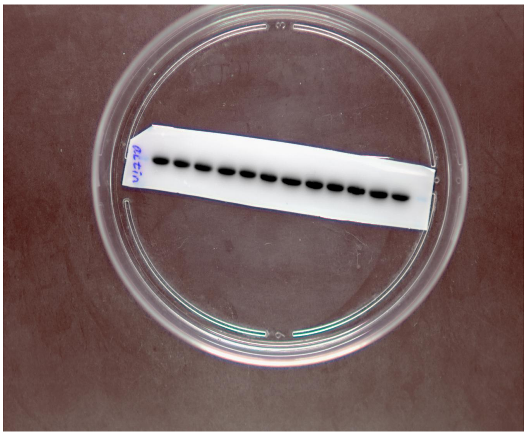

Supplementary Figure 4: Uncropped blots for Figure 3.C

p-ErBb4

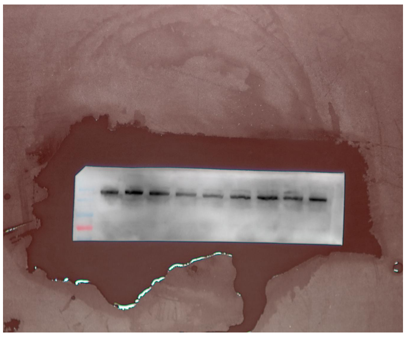

ErbB4

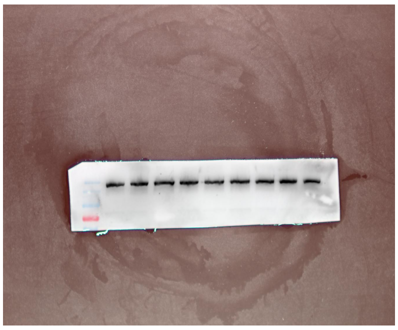

β-Actin

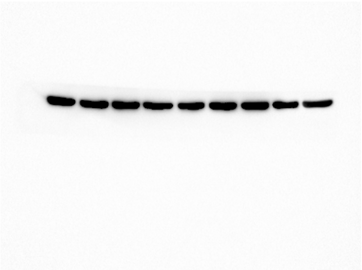

Supplementary Figure 5: Uncropped blots for Figure 5.B

p-ErBb4

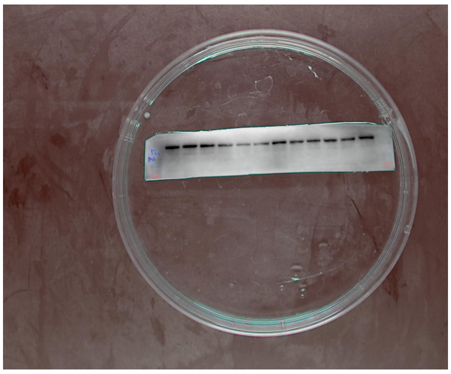

ErbB4

β-Actin

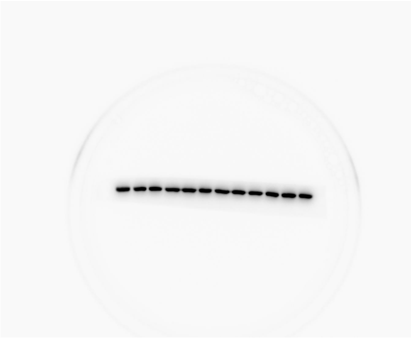

Supplementary Figure 6: Uncropped blots for Figure 5.C

p-ErBb4

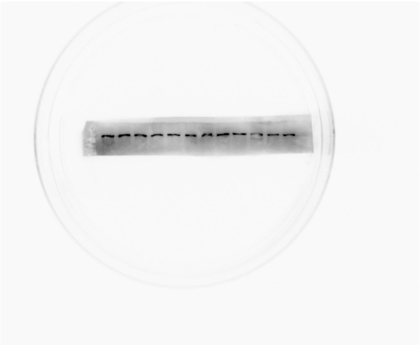

ErbB4

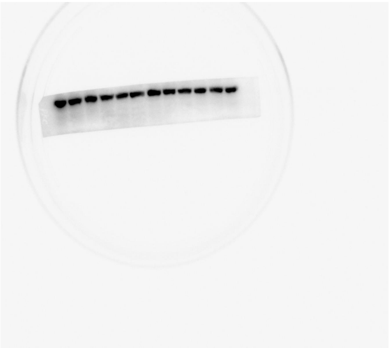

GAD67

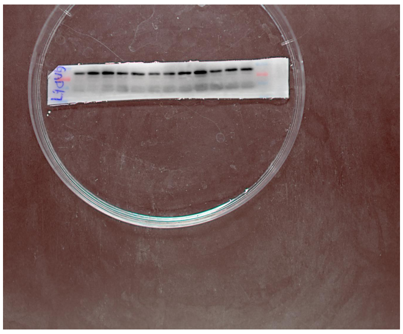

VGAT

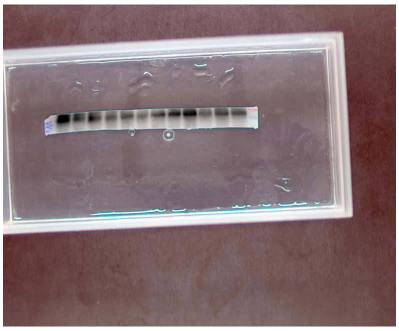

VGluT1

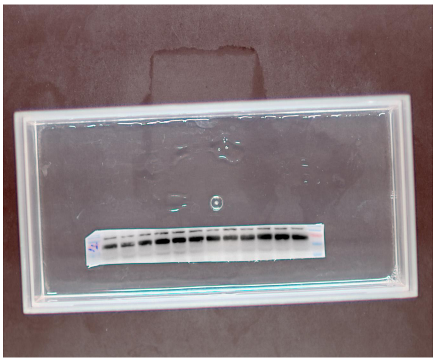

VGluT2

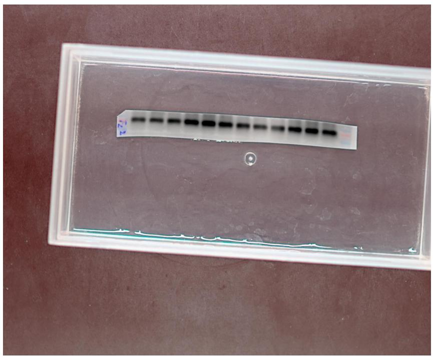

β-Actin

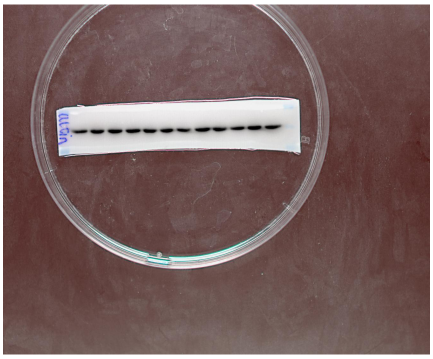

Supplementary Figure 7: Uncropped blots for Figure 5.I

PKCα

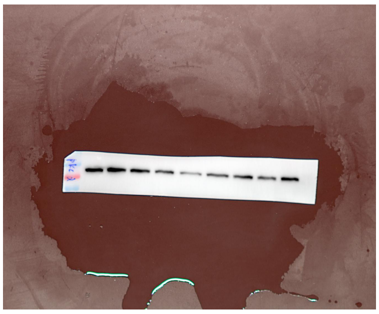

p-MARCKS

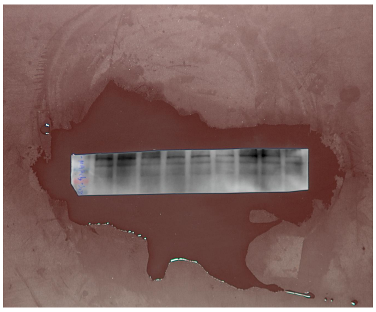

MARCKS

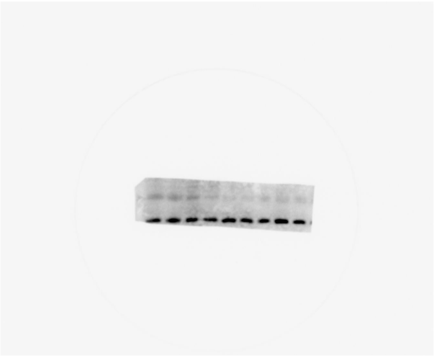

β-Actin

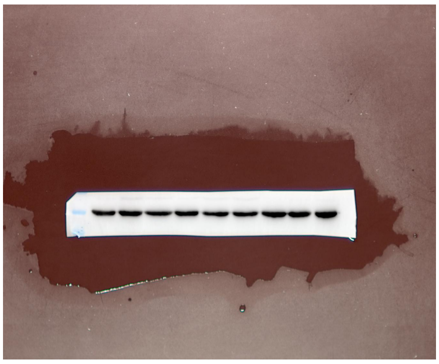

Supplementary Figure 8: Uncropped blots for Figure 6.A

PKCα

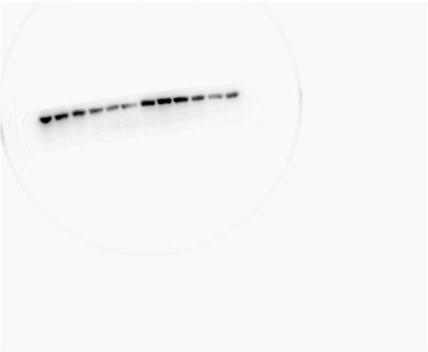

p-MARCKS

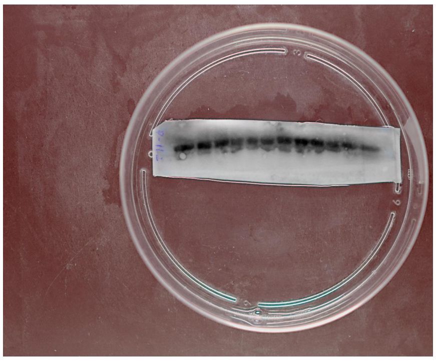

MARCKS

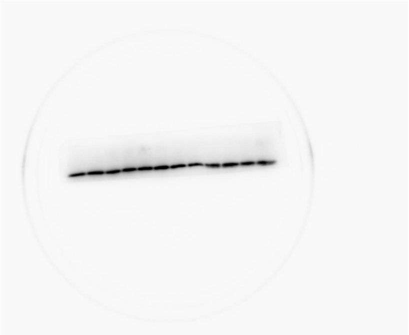

p-ErBb4

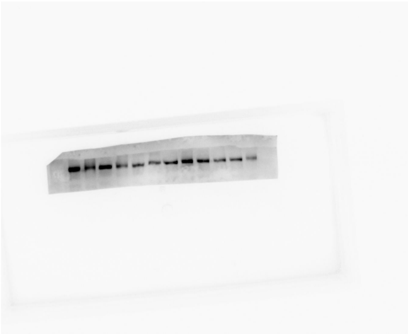

ErbB4

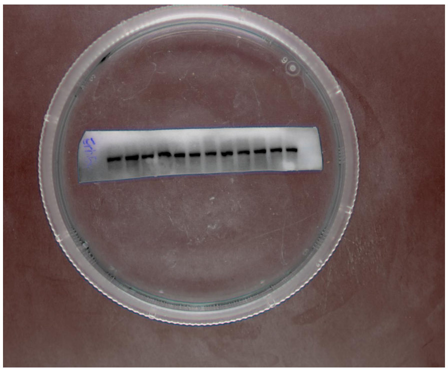

β-Actin

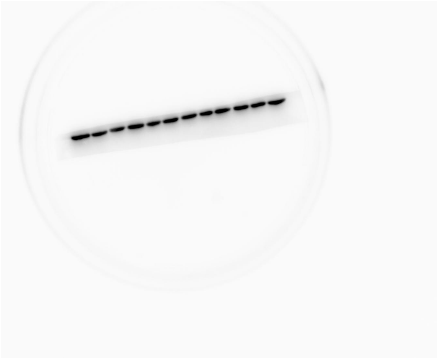

Supplementary Figure 9: Uncropped blots for Figure 6.G

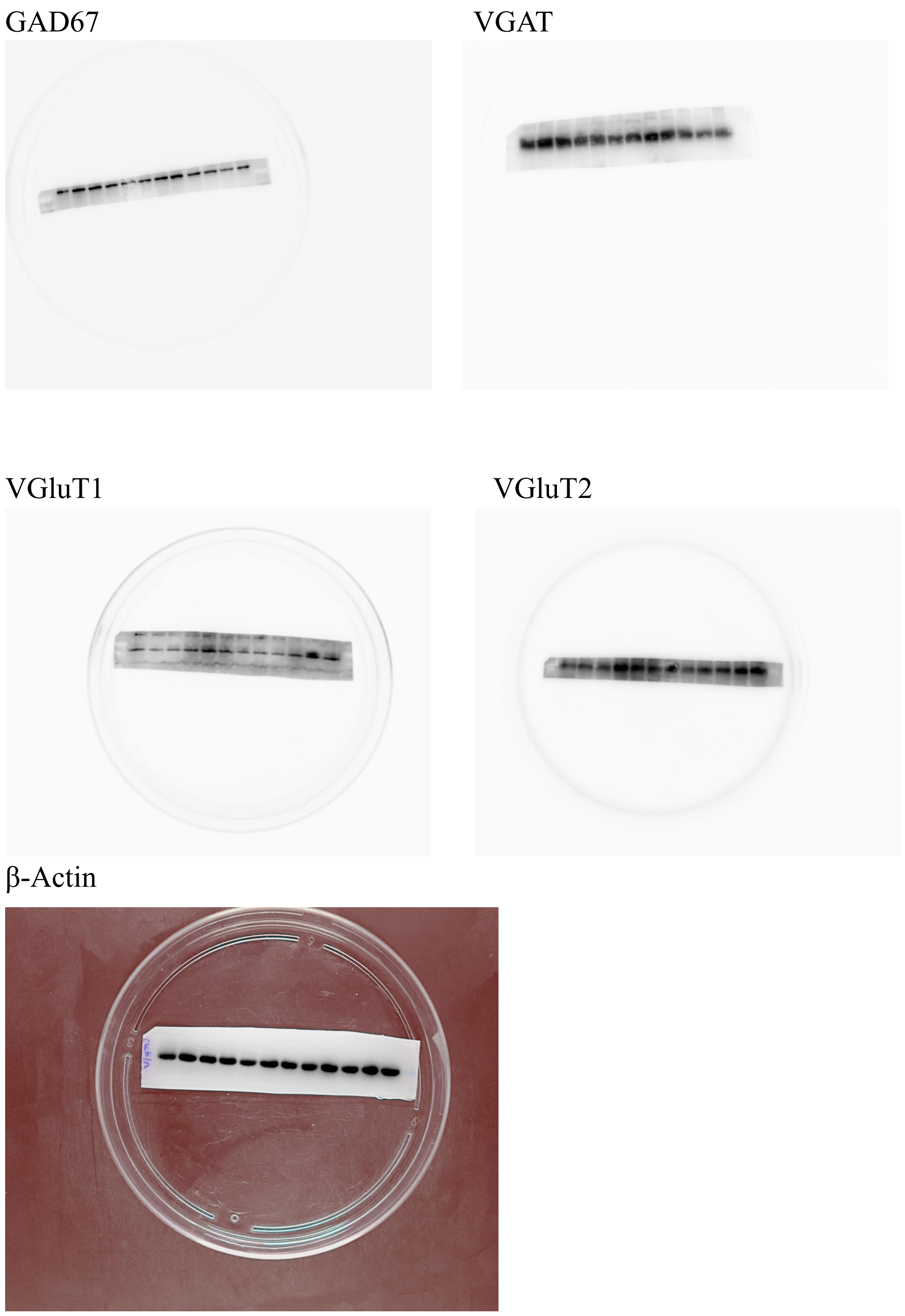

Supplementary Figure10: Uncropped blots for Figure 6.H
